# Roles of the vagina and the vaginal microbiota in urinary tract infection: evidence from clinical correlations and experimental models

**DOI:** 10.3205/id000046

**Published:** 2020-03-26

**Authors:** Amanda L. Lewis, Nicole M. Gilbert

**Affiliations:** 1 Washington University School of Medicine, Department of Molecular Microbiology, St. Louis, United States; 2 Washington University School of Medicine, Department of Obstetrics and Gynecology, St. Louis, United States

## Abstract

Mounting evidence indicates that the vagina can harbor uropathogenic bacteria. Here, we consider three roles played by the vagina and its bacterial inhabitants in urinary tract infection (UTI) and urinary health. First, the vagina can serve as a reservoir for *Escherichia coli*, the most common cause of UTI, and other recognized uropathogens. Second, several vaginal bacterial species are frequently detected upon urine culture but are underappreciated as uropathogens, and other vaginal species are likely under-reported because of their fastidious nature. Third, some vaginal bacteria that are not widely viewed as uropathogens can transit briefly in the urinary tract, cause injury or immunomodulation, and shift the balance of host-pathogen interactions to influence the outcomes of uropathogenesis. This chapter describes the current literature in these three areas and summarizes the impact of the vaginal microbiota on susceptibility to UTI and other urologic conditions.

## Summary of findings

1. Data from clinical studies and model systems highlight the vaginal microbiota as a key factor impacting susceptibility to UTI and other urologic conditions.
2. The vagina can serve as a reservoir for uropathogen colonization.
3. Certain members of the vaginal microbiome are frequently detected in urine yet are underappreciated as uropathogens. Other vaginal bacterial species are likely underreported because they are difficult to culture or to identify.
4. The urinary tract can be transiently exposed to vaginal bacteria, some of which can cause injury to the bladder epithelium and impact pathogenesis of recognized uropathogens.

## 1 Introduction

It is well known, that women suffer from urinary tract infections (UTI) much more frequently than men. Mounting evidence indicates that the vagina can harbor uropathogenic bacteria. According to the current literature we consider the evidence from clinical correlations and experimental models that the vaginal microbiota impacts susceptibility to UTI and other urologic conditions. 

## 2 Methods

We performed literature searches in PubMed. For our overview of the vaginal microbiome and its impact on the urinary tract, we searched “vaginal microbiome” OR “vaginal microbiota” AND “bacterial vaginosis” OR “BV” OR “dysbiosis”. To find articles relating to vaginal colonization by uropathogens, we searched “*Escherichia coli*” OR “*Staphylococcus*” OR “group B *Streptococcus*” OR “GBS” AND “vagina” OR “vaginal”. To identify articles relating to vaginal species that may be underappreciated uropathogens, we examined the list of organisms commonly detected in vaginal microbiome studies and searched these organisms along with “urinary tract” OR “urine” OR “UTI”.

## 3 Results and discussion

In order to better understand the role of the vaginal microbiota in UTI, we consider the “healthy” or “normal” state in reproductive age women and in pregnancy and three roles played by the bacterial inhabitants of the vagina in UTI and urinary health. First, the vagina can serve as a reservoir for *Escherichia coli*, the most common cause of UTI, and other recognized uropathogens. Second, several vaginal bacterial species are frequently detected upon urine culture but are underappreciated as uropathogens, and other vaginal species are likely under-reported because of their fastidious nature. Third, some vaginal bacteria that are not widely viewed as uropathogens can transit briefly in the urinary tract, cause injury or immunomodulation, and shift the balance of host-pathogen interactions to influence the outcomes of uropathogenesis. 

### 3.1 Vaginal microbiota composition

**Figure 1:**
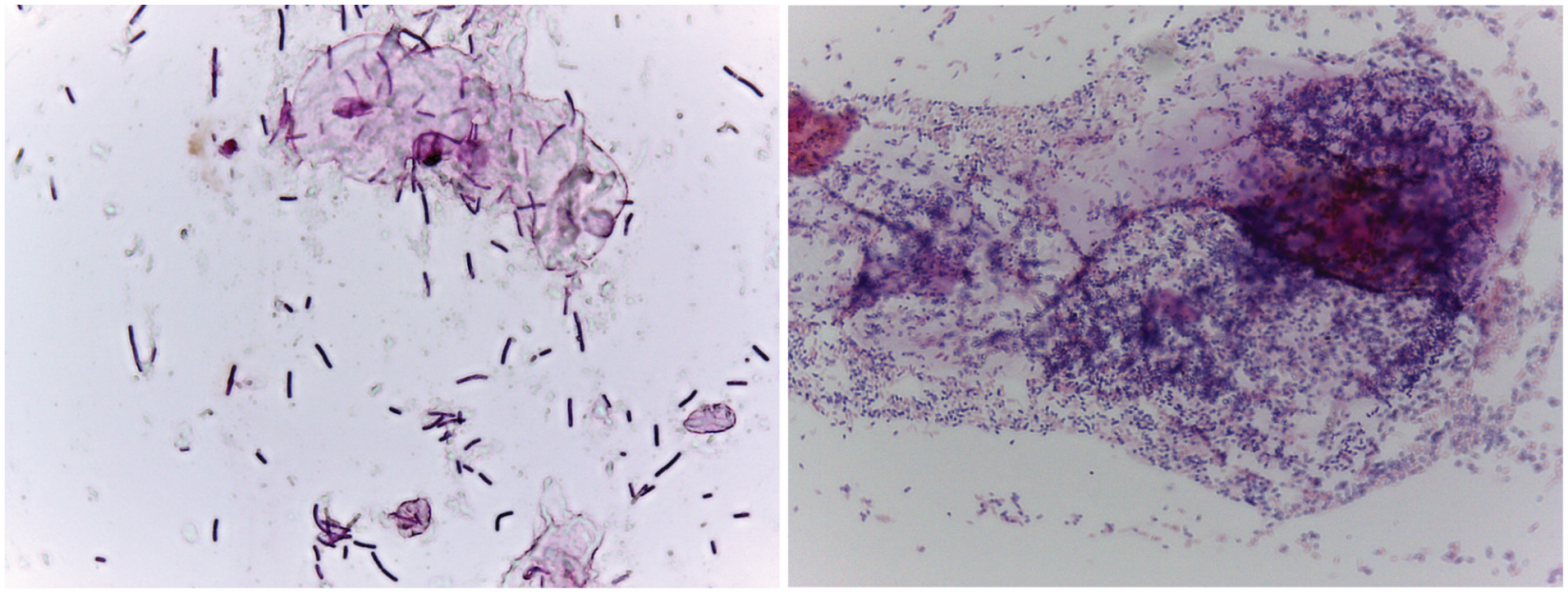
Examples of Gram-stained slides from women with (right, Nugent score = 9) and without (left, Nugent score = 1) BV. In women with a lactobacilli-dominated microbiome (left), long purple rods are the main morphotype. In contrast, women with BV tend to have a lot more bacteria overall, with a significant fraction that do not stain as Gram-positive (i.e. pink). Morphotypes in BV tend not to be long Gram-positive rods. Although not part of the Nugent scoring system, women with BV tend to have large conglomerations of bacteria associated with epithelial cells as seen in the upper right quadrant.

In reproductive age women, the vaginal microbiota is dominated by a few *Lactobacillus* species, including *L. crispatus*, *L. gasseri*, *L. jensenii*, and *L. iners*. These lactobacilli are thought to prevent growth of potential pathogens by maintaining the vagina’s characteristic low pH (by producing lactic acid [1]) and by producing antimicrobials such as hydrogen peroxide and bacteriocin-like substances [2]. Although a *Lactobacillus*-dominant vaginal microbiota is considered the “healthy” or “normal” state, a large proportion of reproductive age women (up to one-third in the United States) have a more diverse vaginal microbiota that contains no or low levels of *Lactobacillus* and a mixture of Gram-negative anaerobes, Actinobacteria, and other *Firmicutes* [3]. This apparent dysbiosis is called bacterial vaginosis (BV) or community state type IV [4]. While fewer studies have examined postmenopausal women, these echo what has been observed in younger cohorts, with some women having *Lactobacillus*-dominant and others having a more diverse polymicrobial vaginal microbiome [5], [6], [7]. A few studies have suggested that pregnancy may favor a stable *Lactobacillus*-dominant vaginal microbiome, however, a diverse vaginal microbiome is nonetheless present in some pregnant women [8]. In the clinic, a woman is diagnosed with BV if she has three of the four following signs (Amsel criteria): vaginal pH>4.5, ‘thin’ grayish homogenous vaginal fluid, vaginal fluid that produces a fishy odor when treated with potassium hydroxide, and the presence of epithelial cells studded with bacteria (i.e., ‘clue-cells’) in wet mount. In the laboratory, BV is diagnosed via the Nugent scoring system [9], which is based on morphotype assessment of Gram-stained slides; a score of 7 or higher on a 10-point scale indicates BV (see Figure 1 (Fig. 1) for examples of Gram-stained slides from women with and without BV). Women with BV are at higher risk of experiencing a wide array of negative health outcomes, including increased risk of a variety of secondary infections and adverse pregnancy outcomes. 

### 3.2 Relationships between the vaginal microbiota, UTI, and urinary health

Data from multiple clinical studies suggest that a woman’s vaginal microbiota affects her susceptibility to UTI [10]. For example, women with BV have higher UTI risk than women with lactobacilli-dominated vaginal microbiota [11], [12], [13]. Furthermore, clinical trials suggest that vaginal interventions that affect the microbiota (e.g., vaginal probiotic and estrogen treatments) can protect against additional episodes of recurrent UTI (rUTI) [14], [15]. As described later in this chapter, many of the bacterial genera found in the BV-associated vaginal environment have been detected, via both culture-dependent and -independent methods, in the urinary tract. Additionally, some of these bacteria have been implicated as causes of acute UTI or other urologic conditions. These findings suggest that fastidious BV-associated organisms may be important in the etiology of uropathology and uropathogenesis; however, in many cases additional studies are needed to ascribe causal roles for these organisms in the urinary tract.

**Figure 2:**
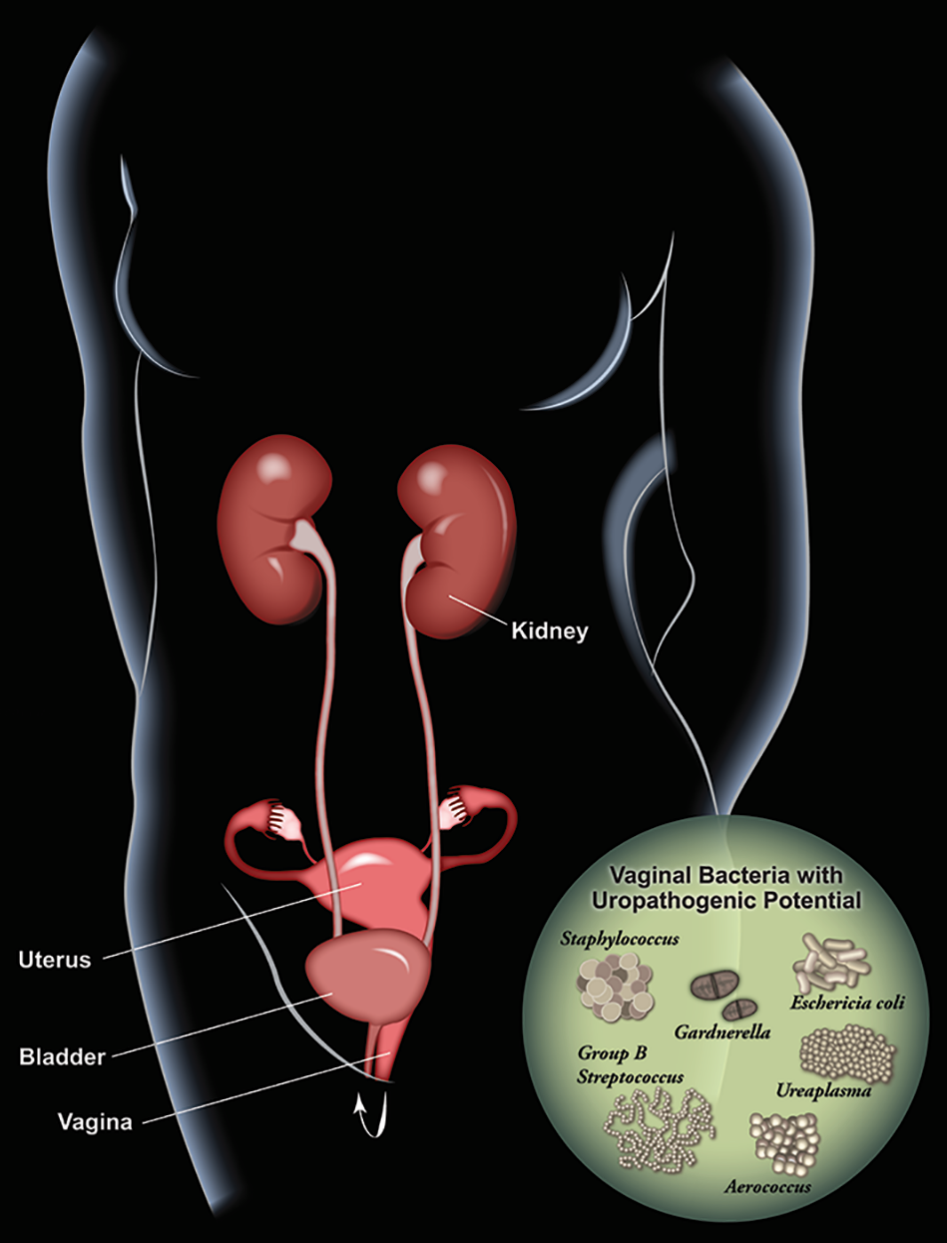
Schematic illustrating vaginal bacteria with potential to impact the urinary tract. The vagina can serve as a reservoir for several bacterial species known to be causes of UTI (*E. coli*, GBS, *Staphylococcus*) as well as underappreciated potential uropathogens (G.* vaginalis*, *Aerococcs* *Ureaplasma*) that can cause UTI and have been associated with urological conditions such as urgency incontinence and “sterile” pyuria.

Why women with a disrupted vaginal microbiota are at increased risk of UTI is unclear. Below, we describe three ways the vaginal microbiota could influence UTI and other urologic conditions. First, the vaginal introitus appears to be a key reservoir for uropathogenic *E. coli*. Second, other somewhat less common uropathogens can also be commonly harbored in the vagina (see Figure 2 (Fig. 2)). Finally, transient exposures of the urinary tract to certain vaginal bacteria may prime the urinary tract for uropathogens or trigger rUTI via other mechanisms.

### 3.3 The vagina provides a reservoir for uropathogenic E. coli and other recognized uropathogens

#### 3.3.1 E. coli

The most common cause of UTI in most patient populations is uropathogenic *E. coli* [16], [17]. The pathogenesis of *E. coli* UTI is often described as a series of colonization events, starting in the gastrointestinal tract, followed by the vaginal introitus and urethral meatus, and finally the bladder and possibly kidneys [10], [18]. This progression makes sense given the proximity of the urethra to the vaginal introitus and anus in females and that longitudinal examinations have indicated that introital and urethral colonization precedes UTI symptom onset [19], [20]. Moreover, multiple clinical studies have found that women with a history of rUTI more commonly have *E. coli* in the vaginal introitus or vagina than do healthy controls [19], [20], [21], [22]. One such study found that *E. coli* colonization of the vaginal introitus reached higher levels in women with a history of UTI (>10^5^ colony forming units/mL) than in controls (<200 cfu/mL) [20]. In a separate study, concurrent colonization of the vaginal introitus and urinary tract by the same strain of *E. coli* (based on random amplified polymorphic DNA fingerprinting) occurred in 85% of paired isolates from women with a history of rUTI [20]. Together, these results demonstrate that the vagina provides a reservoir for uropathogenic *E. coli*. 

As described above, *Lactobacillus* in the vagina may prevent colonization by potential pathogens such as *E. coli*. Indeed, women with low levels of lactobacilli more commonly carry vaginal *E. coli* than do those with lactobacilli-dominated microbiomes [13], [21]. In one study, *E. coli* vaginal (introital) colonization was more common among women whose vaginal introitus was negative for hydrogen peroxide-forming lactobacilli than among those whose introitus was positive for these ‘beneficial’ bacteria (35% vs. 11%, odds ratio [OR], 4.0; P=0.01) [21]. Multiple studies in both nonpregnant and pregnant women suggest that those with BV have increased risk for UTI (odds ratios from 2.21 to 13.75) [12], [23], [24]. In one study, BV was associated with both *E. coli* introital colonization and UTI [13]. In another study, over 50% of women with a history of UTI, but only 13% of those with no UTI history, had abnormal vaginal microbiota (*P*=0.03) as defined by a Nugent score greater than 3 (mean 4.6 vs. 1.7). Having a Nugent score of 7 or greater (indicative of BV) was also more common in UTI-prone women (6/22 [27%] vs. 1/17 [6%], *P*=0.095) [25]. This may be because lactic acid, hydrogen peroxide, and other small molecules produced by lactobacilli create a hostile environment for potential pathogens, including uropathogens, in the vagina [26]. Additional studies in experimental models are needed to define the effects of specific small molecules on the vaginal environment. 

One intervention that appears to restore *Lactobacillus* colonization and may protect against rUTI in some patients is vaginal probiotics. A double-blind placebo-controlled trial studied 100 premenopausal women with at least one UTI in the past 12 months. Women received either intravaginal suppositories of powdered *L. crispatus* (Lactin-V) once daily for 5 days and then weekly for 10 weeks or placebo suppositories at the same interval. Fewer women who established high-level vaginal *L. crispatus* colonization (≥10^6^ throughout follow-up) developed rUTI (relative risks were 0.07 for Lactin-V and 1.1 for placebo; *P*<0.01) [14]. In contrast, a smaller study found that vaginal application of other types of lactobacilli (*L. casei* and *L. rhamnosus*) twice weekly did not reduce the UTI incidence [27]. However, the latter study used a higher *E. coli* threshold (10^4^ vs. 10^2^) to diagnose an rUTI. Additionally, *L. casei* and *L. rhamnosus*, which are more common in the intestine than in the vagina, failed to colonize the vagina [27]. Future studies are needed to develop effective vaginal prebiotic and probiotic strategies to treat or prevent rUTI.

Another promising intervention is vaginal estrogen. This idea is based on the observation that high levels of vaginal lactobacilli occur only in 25% to 30% of postmenopausal women (who tend to have low estrogen levels) but in 60% to 70% of women who receive estrogen replacement therapy. In a placebo-controlled study of postmenopausal women with rUTI, vaginal estrogen restored vaginal *Lactobacillus* colonization and decreased vaginal *Enterobacteriaceae* colonization (67% of women pre-treatment vs. 31% post-treatment). Moreover, the UTI incidence was lower in the estrogen group than in the placebo group (0.5 vs. 5.9 episodes per patient year; *P*<0.001) [15]. In a separate study, women receiving vaginal estrogen had a 45% cumulative likelihood of remaining UTI-free over a 36-week follow up period, whereas those in the control group had only 20% likelihood of remaining UTI-free [28]. For a more detailed description of the clinical studies examining the role of *Lactobacillus* and *E. coli* vaginal colonization and vaginal interventions for UTI, please see this recent review [10]. 

#### 3.3.2 Staphylococcus

The most frequent Gram-positive agent of community-acquired UTI is *S. saprophyticus*, but *S. aureus* and *S. epidermidis* can also cause UTI in certain settings (catheterization, pregnancy) [29], [30], [31]. *Staphylococcus* uropathogenesis was recently reviewed [32]. Data from *in vitro* studies and rat UTI models have revealed several factors required for *S. saprophyticus* virulence, including the secreted surface-associated proteins Aas (hemagglutinin) and Ssp (lipase); the cell wall proteins UafA, SdrI, SssF, and UafB, which mediate adherence; and a urease that is associated with urinary stone formation. Additionally, data from a mouse model suggest that the nickel ABC-transporters Opp2 and Opp5a contribute to *S. aureus* uropathogenesis. 

Both *S. aureus* and *S. epidermidis* have been detected in vaginal or cervical samples [33], [34], [35]. Vaginal *S. aureus* has been implicated in toxic shock syndrome and aerobic vaginitis [36], [37], [38], a controversial inflammatory condition often mistaken for BV or *Candida* yeast infection. One study found that women with vaginal toxicogenic *S. aureus* were significantly more likely than those without *S. aureus* to harbor vaginal *E. coli* [38]. However, vaginal colonization by staphylococci has not been examined as a risk factor for UTI.

#### 3.3.3 Group B Streptococcus

*Streptococcus agalactiae*, otherwise known as group B *Streptococcus* (GBS), is a Gram-positive β-hemolytic chain-forming coccus that commonly inhabits the lower gastrointestinal tract and the vagina. Although GBS colonization is often asymptomatic, GBS has been implicated in aerobic vaginitis [39]. GBS is also a recognized uropathogen, causing 2–3% (or ~160,000 cases annually in the U.S.) of all uncomplicated UTIs [17], [40]. GBS UTIs are more common in certain vulnerable populations. For example, among nursing home residents over 70 years of age, up to 39% of UTI cases involve GBS [41]. Additionally, GBS often causes asymptomatic bacteriuria and is found at significant titers in up to 7% of pregnant women [42], [43]. GBS is also frequently found in the urinary tract of people with diabetes, immunocompromised individuals, and those with pre-existing urologic abnormalities, all of whom are at increased risk of ascending pyelonephritis that can progress to bacteremia and/or urosepsis [44], [45]. Although GBS can inhabit both the vagina and the urinary tract, no studies have assessed the association between vaginal GBS and GBS UTI.

Mouse models of GBS UTI [46], [47], [48], [49] have mainly used serotype III GBS strains, which cause more symptomatic UTIs than most other serotypes [45]. The role of GBS virulence factors has not been as thoroughly examined in the urinary tract as in other niches, such as the bloodstream. However, available data suggest that the β-hemolysin/cytolysin is dispensable, whereas sialic acid residues of the GBS capsular polysaccharide are important for GBS survival in the urinary tract [47], [48]. Further studies are needed to define the bacterial and host mechanisms governing GBS disease in the urinary tract. 

### 3.4 Other vaginal bacterial species that are underappreciated as uropathogens

#### 3.4.1 Gardnerella vaginalis

*Gardnerella vaginalis*, a facultative Gram-variable *Actinobacteria* (a class frequently called ‘high-GC Gram-positives’), is best known as a frequent isolate and dominant member of the vaginal microbiota in BV. Although *G. vaginalis* is considered an unusual primary pathogen of the urinary tract, this bacterium can cause acute UTI [50]. The contribution of *G. vaginalis* to urinary tract pathology is likely underestimated for two reasons. First, *G. vaginalis* does not grow under the standard (aerobic) culture conditions that most clinical microbiology labs use. Second, when labs identify *G. vaginalis* in urine cultures, they do not report it as a potential uropathogen (even if present in pure culture at levels exceeding clinical thresholds for UTI diagnosis). Several studies suggest that *G. vaginalis* should be considered as a potential cause of urinary tract pathology. For example, in one study using appropriate culture conditions to detect it, *G. vaginalis* was isolated from 2.3% of urines from hospitalized patients, often in pure culture and >10,000 colony forming units per milliliter (cfu/ml). Compared to individuals in whom *G. vaginalis* was not detected, patients with *G. vaginalis* bacteriuria were more likely to have a history of rUTI or current pyelonephritis (kidney infection) [51]. These patients often reported symptoms, and 58% had pyuria (neutrophils in urine) [51]. Another study showed that the frequency of *G. vaginalis* in catheterized urine samples was higher in women with urgency urinary incontinence than in women with other urologic conditions [52]. Other studies have used suprapubic needle aspiration to collect urine from the bladder, bypassing possible vaginal contaminants, concluding that pregnancy increases women’s risk of harboring *G. vaginalis* in their bladders and that *G. vaginalis* was especially common in women with underlying renal disease [53], [54], [55], [56], [57]. In another study, *G. vaginalis* was commonly found in suprapubic aspirates from women with reflux scarring and “sterile pyelonephritis” [58]. Bladder washout studies strongly suggested that *G. vaginalis* was present in the kidneys of 75% of these patients. Finally, case reports implicate *G. vaginalis* in more serious diseases. For example, *G. vaginalis* has been isolated from women's bloodstreams during or after giving birth [59], and *G. vaginalis* bacteremia has been implicated in systemic diseases coinciding with urolithiasis (kidney stones) [60]. In short, *G. vaginalis* has been implicated in rUTI, urgency incontinence, kidney disease, and systemic infections originating in the genitourinary system. 

Thus far, few reports have assessed *G. vaginalis* in both the vagina and the urinary tract. However, one recent study compared the microbiota of paired midstream urine and vaginal fluid from 42 women with BV [61] and found high levels of *G. vaginalis* in urine in a subset of women. The authors noted an association between urine and vaginal fluid communities in ~60% of women with urotypes dominated by *G. vaginalis*, *Prevotella amni*, *Atopobium vaginae*, or *Sneathia amnii*. In contrast, they reported only a 10–30% correlation among other urotypes (*E. coli*, *Lactobacillus*, etc.). The authors state that urine and vaginal microbiota patterns were not correlated in most women, and suggest that the two communities are therefore distinct in many women. However, despite the suggestion that some vaginal bacteria, including *G. vaginalis*, can exist concurrently in the vagina and urinary tract, this manuscript does not appear to have evaluated the co-occurrence of individual taxa per se in the two different compartments. The findings raise a number of interesting questions, including whether vaginal colonization with *G. vaginalis* a risk factor for G. vaginalis in urine or having concurrent or subsequent urologic pathologies. *G. vaginalis* isolates have highly diverse genomes and can be divided into at least four clades. In fact, some authors have suggested that *G. vaginalis* should be divided into multiple species or even genera [62], raising the question of whether certain *G. vaginalis* subtypes are more likely to be pathogenic in the urinary tract. 

#### 3.4.2 Aerococcus

*Aerococcus* is an α-hemolytic and microaerophilic or facultatively anaerobic Gram-positive coccus. Several *Aerococcus* species, including *A. urinae*, *A. viridans*, and *A. sanguinicola*, can cause UTI and urosepsis [63], [64], [65], [66], [67], [68], [69]. Patients with *Aerococcus* UTI often have underlying risk factors, such as urological abnormalities or older age [66], [67], [70]. Many case reports describing significant *Aerococcus* titers in urine also reported isolating the bacterium from blood [66], [71], [72]. *Aerococcus* must be properly identified and treated to avoid life-threatening systemic infection [68]. However, *Aerococcus* is difficult to distinguish from viridans-group streptococci with routine phenotypic tests and thus requires molecular tools such as amplification and 16S rRNA sequencing, a method not commonly employed in clinical microbiology labs. Additionally, most reported *Aerococcus* isolates are resistant to sulfonamides [69], [70]. Thus, when *Aerococcus* is not recognized, ineffective antibiotic treatment can be given, leading to rapid progression to systemic infection [67], [69], [71], [72], [73]. In addition to being implicated in UTI, *Aerococcus* was more frequently cultured from urine (collected by transurethral catheterization) from patients with urgency urinary incontinence than from women with other urinary tract conditions [52]. To our knowledge, *Aerococcus* pathogenesis in the urinary tract has not been examined with *in vivo* models. 

*Aerococcus sp*. are commonly isolated from the human vagina and urinary tract and from air, dust, and vegetation. More studies are needed to understand how these natural *Aerococcus* niches affect pathogenesis and the clinical course of infections [74]. *Aerococcus* vaginal colonization is more common and abundant in women with BV [75], [76], but no studies have examined whether women with BV, or their sexual partners, are at increased risk for *Aerococcus* UTI.

#### 3.4.3 Ureaplasma

*Ureaplasma* species, including *U. urealyticum* and *U. parvum*, are characterized by their ability to hydrolyze urea. *U. parvum* UTI pathogenesis was examined in Fischer 344 rats, revealing two distinct outcomes. Some animals developed a minimal immune response with limited monocytic and lymphocytic lesions and elevated urinary interferon (IFN)-γ, interleukin (IL)-18, and monocyte chemoattractant protein-1. Other animals developed an exaggerated pro-inflammatory immune response with elevated urinary IL-1α, IL-1β, CXCL1 (a.k.a. KC), neutrophilic lesions with extensive uroepithelial hyperplasia, and struvite (stone) formation [77]. A separate study demonstrated that rat strains differed in their susceptibility to *U. parvum* UTI and struvite formation [78]. Together, these studies suggest that host factors influence *U. parvum* UTI outcomes. 

Between 40% and 80% of sexually active women have vaginal/cervical ureaplasmas [79]. Some studies indicate that *Ureaplasma* vaginal colonization increases during BV, but others have failed to find associations with BV [80] or vaginal symptoms in general [81]. However, ureaplasmas are reproducibly the most common cause of amniotic fluid infection in pregnant women, suggesting the bacteria can invade urogenital tissues and cause serious infection [82], [83]. Although acute cystitis by *Ureaplasma* is rare, the genus has been implicated in lower urinary tract symptoms [84], [85], including overactive bladder [86], “sterile pyuria” [87], and unexplained chronic voiding symptoms [85]. Further studies are needed to determine whether vaginal *Ureaplasma* is a risk factor for UTI or other lower urinary tract symptoms.

### 3.5 Covert pathogenesis

Although many consider the bladder to be sterile in the absence of UTI, clinical microbiology laboratories often find that urines have “insignificant” titers of bacteria (the “significant” threshold is usually ~100,000 cfu/ml). Additionally, numerous reports have described the composition of bacteria in urine (the urinary microbiome), further suggesting that bacteria can exist, at least transiently, in the urinary tract. The urinary microbiome is covered in greater detail elsewhere. Here, it is relevant to note that many of these studies have detected bacterial genera that have been independently found as part of the vaginal microbiota, including *Lactobacillus*, *Streptococcus*, and the BV-associated organisms *Gardnerella*, *Prevotella*, *Bacteroides*, and others [88]. Several studies classified urine samples into “urotypes” based on the dominant organisms, *L. crispatus* and *G. vaginalis* often being the most frequent [52]. Although the presence of these bacteria in urine is often attributed to contamination of urine by vaginal fluid, several studies have identified *Gardnerella* and lactobacilli in urine collected by transurethral catheterization or suprapubic aspiration, which completely rules out the possibility of contamination by periurethral or vaginal bacteria [89]. 

Vaginal bacteria can enter the urinary tract by mechanical transfer from nearby sites [10], [18], such as during sexual activity. In support of this idea, sexual activity (and its frequency) is one of the strongest risk factors for UTI and rUTI [15], [16], [90], [91], [92], [93], [94]. In addition to uropathogens such as *E. coli*, sexual activity likely also transfers numerous other vaginal bacteria. This possibility points to a *new idea*: that transient urinary tract exposure to certain vaginal bacteria can directly influence UTI pathophysiology even if these vaginal bacteria do not colonize the bladder or are cleared by the host before UTI diagnosis. This idea has been referred to as “covert pathogenesis” and is supported by findings from mouse models in which the urinary tract was exposed to different common vaginal bacteria within the context of *E. coli* UTI. For example, in one model, mice were exposed to group B Streptococcus (GBS, see above), a known immunomodulatory bacterium during acute or chronic bladder lumen infection by *E. coli* in C3H/HeN mice. The studies demonstrated that the presence of GBS enhanced *E. coli* survival in the bladder lumen in the early hours of acute infection, despite the fact that GBS was more rapidly cleared by the host during *E. coli* infection. In fact, even attenuated strains of *E.c.* lacking the ability to adhere to the bladder epithelium due to mutation of the type I pilus, had higher *E. coli* bladder lumen titers when GBS was present [11]. The presence of GBS also had effects on *E. coli* infection during chronic inflammatory infection of the bladder lumen by *E. coli*. This study provided an initial proof of principle that the composition of bacterial exposures to the urinary tract (containing *E. coli*) may influence initial host-*E. coli* interactions and therefore help determine whether *E. coli* causes UTI.

**Figure 3:**
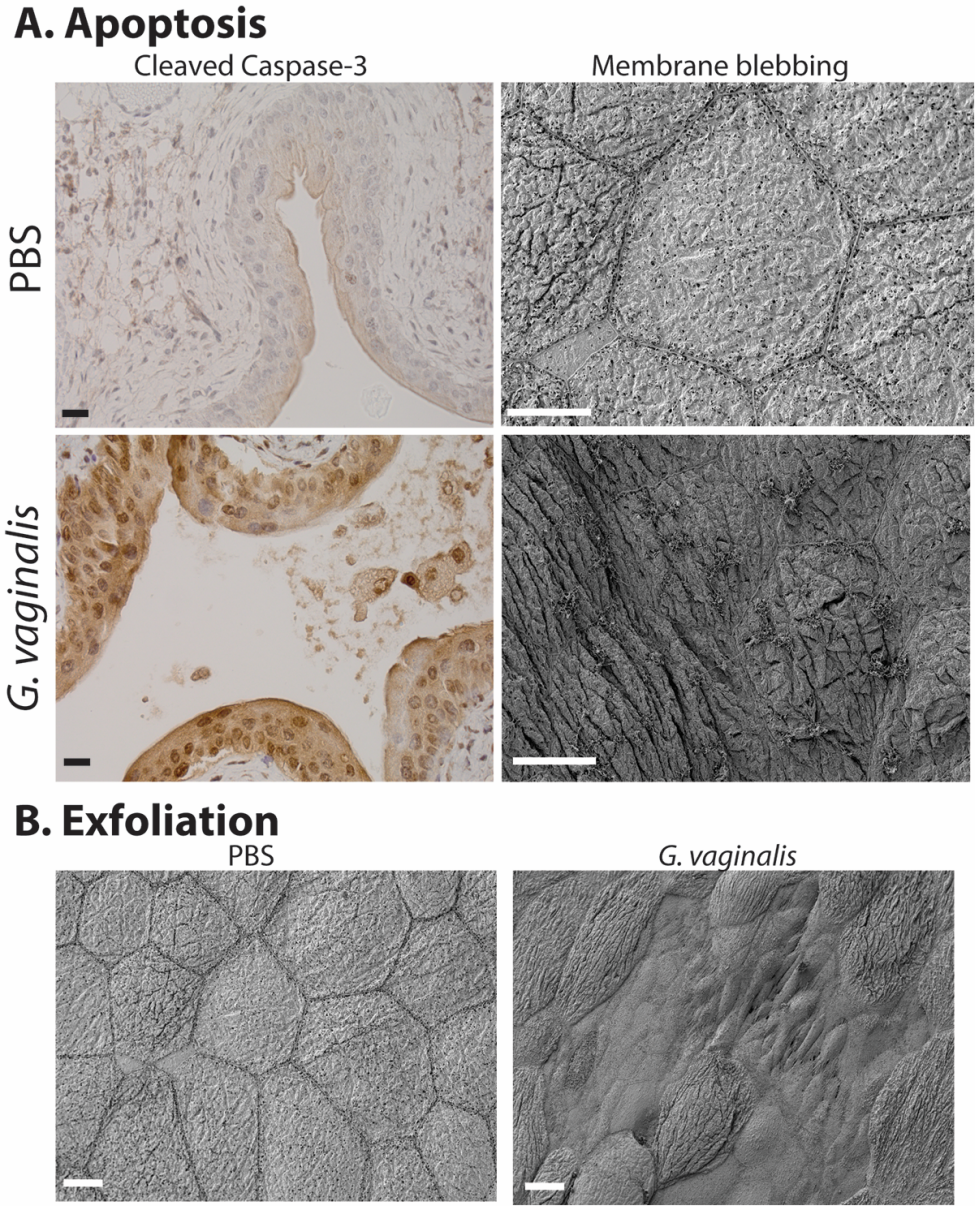
Gardnerella vaginalis induces apoptosis (A) and exfoliation (B) of the mouse bladder epithelium following two transurethral exposures, using our previously reported model [95]. Our blinded observation showed that ~75% of animals exposed to Gardnerella (compared to only ~25% of controls) exhibited staining for cleaved caspase-3, a marker of apoptotic cell death, in the outermost epithelium (A, left). Membranous protrusions were another feature we observed by scanning electron microscopy that is consistent with apoptosis (A, right). We also observed that G. *vaginalis* triggers exfoliation of superficial umbrella cells, revealing smaller underlying cells of the transitional epithelium (B). Scale bars = 20 μm.

In another model, effects of another vaginal bacterium were studied during latent *E. coli* infection within intracellular epithelial reservoirs in C57/Bl6 mice (from a previous experimental infection), to investigate possible triggers of recurrent *E. coli* UTI arising from such reservoirs [95]. In these mice, two exposures to *G. vaginalis* triggered *E. coli* emergence into the bladder lumen, resulting in rUTI. *G. vaginalis* exposure also increased the incidence of severe *E. coli* kidney infections. *G. vaginalis* caused these effects despite being rapidly cleared from the urinary tract (by 12 hours in most mice). Even in the absence of latent *E. coli* (but also in the rUTI model), *G. vaginalis* caused apoptosis and exfoliation of the bladder epithelium (see Figure 3 (Fig. 3)) and also caused kidney damage by an IL-1-receptor-mediated mechanism. These findings suggest that *G. vaginalis* could be an important trigger of rUTI and a risk factor for pyelonephritis in women. If data from clinical studies support this idea, then new treatment options (antibiotics to limit *G. vaginalis* colonization) could be tested to help prevent rUTI, especially among patients with BV. 

## 4 Further research

We have described multiple mechanisms by which vaginal bacteria can impact UTI incidence or pathogenesis. Vaginal interventions, whether to eliminate the uropathogen reservoir or to treat associated vaginal conditions (e.g., BV), should be investigated as means to improve UTI outcomes, particularly in patients with rUTI for whom chronic antibiotic treatment is the only other option. 

## 5 Conclusions

Although *E. coli* is unquestionably the dominant cause of UTI in young, sexually active women, it is clear that UTI and recurrent UTI stem from a wide array of etiologies. As outlined here, vaginal bacteria may cause UTI, either themselves (i.e. a traditional uropathogen using the vagina as a reservoir) or by acting as a “covert pathogens” to facilitate pathogenesis of another organism. Clinical microbiology labs commonly designate vaginal bacteria – whether identified in pure culture or detected alongside an accepted uropathogen that is present at levels above the usual clinical threshold for UTI – as being of “questionable clinical significance”. We emphasize that, rather than meaning that vaginal bacteria in urine are not important, this simply means that we do not fully understand their effects. UTI caused or encouraged by vaginal bacteria represent significant etiologies that should not be overlooked, as evidenced by findings that vaginal interventions to increase lactobacilli colonization have promising effects in certain patient subsets, as well as experimental models showing that certain vaginal bacteria can be “covert pathogens” (i.e. encourage virulence by recognized uropathogens despite their own rapid clearance). 

## Note

This article is also to be published as a chapter of the Living Handbook “Urogenital Infections and Inflammations“ [96].

## Acknowledgements

We thank Deborah Frank for editorial assistance and thoughtful feedback on the manuscript.

## Competing interests

The authors declare that they have no competing interests.
